# Glycosaminoglycans are involved in bacterial adherence to lung cells

**DOI:** 10.1186/s12879-017-2418-5

**Published:** 2017-05-02

**Authors:** Olga Rajas, Luis M. Quirós, Mara Ortega, Emma Vazquez-Espinosa, Jesús Merayo-Lloves, Fernando Vazquez, Beatriz García

**Affiliations:** 1 Pneumology Service, Hospital La Princesa, Institute for Health Research (IP), Hospital Universitario de La Princesa, Madrid, Spain; 2 University Institute Fernandez-Vega (IUFV), University of Oviedo and Eye Research Foundation (FIO), Oviedo, Spain; 3 Department of Functional Biology, University of Oviedo, Oviedo, Spain; 4 Biobank Coordinator, Institute for Health Research (IP), Hospital Universitario de La Princesa, Madrid, Spain; 5 Department of Microbiology, Hospital Universitario Central de Asturias, Oviedo, Spain

**Keywords:** Glycosaminoglycans, Pathogenic bacteria, Infection, Host interaction, Lung cells

## Abstract

**Background:**

Lower respiratory infections are among the top ten causes of death worldwide. Since pathogen to cell adhesion is a crucial step in the infection progress, blocking the interaction between eukaryotic receptors and bacterial ligands may enable the pathogenesis process to be stopped. Cell surface glycosaminoglycans (GAGs) are known to be mediators in the adhesion of diverse bacteria to different cell types, making it of interest to examine their involvement in the attachment of various pathogenic bacteria to lung cells, including epithelial cells and fibroblasts.

**Methods:**

The function of cell surface GAGs in bacterial adhesion was studied by reducing their levels through inhibiting their biosynthesis and enzymatic degradation, as well as in binding competition experiments with various species of GAGs. The participation of the different bacterial adhesins in attachment was evaluated through competition with two peptides, both containing consensus heparin binding sequences. Blocking inhibition assays using anti-syndecans and the enzymatic removal of glypicans were conducted to test their involvement in bacterial adhesion. The importance of the fine structure of GAGs in the interaction with pathogens was investigated in competition experiments with specifically desulfated heparins.

**Results:**

The binding of all bacteria tested decreased when GAG levels in cell surface of both lung cells were diminished. Competition experiments with different types of GAGs showed that heparan sulfate chains are the main species involved. Blocking or removal of cell surface proteoglycans evidenced that syndecans play a more important role than glypicans. The binding was partially inhibited by peptides including heparin binding sequences. Desulfated heparins also reduced bacterial adhesion to different extents depending on the bacterium and the sulfated residue, especially in fibroblast cells.

**Conclusions:**

Taken together, these data demonstrate that the GAG chains of the cell surface are involved in the adhesion of bacterial adhesins to lung cells. Heparan sulfate seems to be the main species implicated, and binding is dependent on the sulfation pattern of the molecule. These data could facilitate the development of new anti-infective strategies, enabling the development of new procedures for blocking the interaction between pathogens and lung cells more effectively.

## Background

Currently, infectious pathologies remain an important health problem, being among the 10 leading causes of death worldwide [1]. In addition to the infectious diseases not yet eradicated, emerging and re-emerging infections may also appear. This is frequently due to multiple factors including environmental changes, lack of prevention measures, travel and global trade, alterations in host susceptibility and, particularly, adaptive genetic changes in the microorganisms themselves [2]. New adaptations in causative agents provide them with a temporary evolutionary advantage against environmental factors, host defenses and antimicrobial drugs [2, 3], as in the case of some *Staphylococcus aureus* strains, which have acquired resistance to multiple antibiotics, resulting in it becoming the leading cause of chronic infections associated with indwelling medical devices [4].

Of the myriad communicable pathologies currently affecting humankind, the World Health Organization has highlighted the threat from lower respiratory infections and tuberculosis, both of which continue to be among the global top ten causes of death [1]. Although infections of the lower respiratory tract are caused by a variety of pathogens including viruses and fungi, bacteria are the main causative agents [5].

The human body is largely exposed to different bacterial pathogens through the skin and mucous membranes, including the respiratory mucosa [6]. After using a suitable portal of entry, the microorganisms must reach their target site in the body and accomplish the most critical step, the establishment of the focus of the infection. This crucial process implies that bacterial pathogens are capable of adhering to and remaining attached to the cell surface without being dislodged by host defenses [7, 8]. Pathogenic microorganisms have developed diverse virulence factors, and these may cooperate to accomplish the establishment of a pathogen through mediation of the adhesion and colonization phases, through promoting tissue damage and through spreading the pathogen and overcoming the host immune system [7, 8].

Bacterial adhesins need to recognize and interact specifically with host cell surface receptors in order to achieve adequate adherence and colonization [6]. Eukaryotic receptors may also be involved in subsequent stages of the infectious process, including invasiveness, organotropism, and interference in host defense response [7]. A variety of cell surface molecules can act as receptors for microorganisms, including proteins, carbohydrates, lipids, and various different combinations of these.

Proteoglycans (PGs) are a type of glycoconjugate that act as receptors for multiple microbial pathogens [9]. These complex molecules are composed of long unbranched chains of polysaccharides called glycosaminoglycans (GAGs), which are covalently attached to a wide variety of core proteins [10]. These molecules possess a high negative charge, and are formed by repeating units of uronic acid or galactose and an amino sugar, either N-acetyl glucosamine or N-acetylgalactosamine. There are four major classes of GAGs: heparin/heparan sulfate (HP/HS), chondroitin sulfate (CS), keratan sulfate, and hyaluronic acid, the latter being the only one not covalently bound to a core protein [10]. GAGs display remarkable structural diversity, which is the result of interrelated enzymatic reactions, including N- and O- sulfations and epimerization, that occur heterogeneously along the chain [11, 12]. Due to the diversity of core proteins, and especially to the diversity of composition patterns, length, epimerization and sulfation of saccharide chains, the PGs have great heterogeneity, which enables them to fulfil numerous functions. Modifications in GAG chains create specific binding motifs for many ligands, such as cytokines, chemokines, growth factors, enzymes and enzyme inhibitors, and extracellular matrix (ECM) proteins [13–17]. These molecules are also involved in several physiological activities, including organization of the ECM, regulation of proliferation, differentiation and morphogenesis, cytoskeletal organization, tissue repair, inflammation and vascularization [18–20]. In addition, a variety of roles have been ascribed to these molecules in pathological process, including non-infectious pathologies such as cancer [21, 22] as well as infectious pathologies generated by diverse pathogens [23, 24]. Different types of pathogen recognize GAGs as receptors, including a wide spectrum of viruses, bacteria and parasites [24–26].

In addition to their remarkable heterogeneity, PGs are ubiquitous, and are widely distributed on cell surfaces, in pericellular locations and in cytoplasmic secretory vesicles [10, 13]. The expression and composition of GAGs are variable, depending on the cell type and the physiological conditions [25]. These features make PGs excellent candidates as host receptors for microorganisms, with microbial pathogens showing a preference for interacting with HS chains attached to cell surface proteoglycans (HSPGs), and principally with respect to two families: the transmembrane syndecans (SDCs) and the glycosylphosphoinositide-linked glypicans (GPCs), which have four and six members respectively [26, 27].

GAGs play significant roles in many pathophysiological processes in the ECM of the lung: regulating hydration and water homeostasis, maintaining structure and function, modulating the inflammatory response, and influencing tissue repair and remodeling [28]. Many studies have described the roles played by PGs in a wide range of pulmonary diseases, including malignant mesothelioma, pulmonary edema, fibrosis, asthma, emphysema, and bronchiectasis [28–31].

The aim of this article is to investigate the involvement of PGs and their GAG chains as receptors for certain common respiratory bacterial pathogens. The study analyzes the role played by different molecular species of PGs in bacterial adherence, as well as the importance of specific molecular aspects of GAG chain structure on their interaction with microorganisms, particularly the influence of sulfation of saccharide chain residues. The analysis was carried out using both epithelial cells and fibroblasts of pulmonary origin since different profiles of cell surface GAGs are to be expected for the two cell types. Ultimately, understanding the complexity of the adhesion process would allow for the possibility of bacterial infections to be controlled via preventing the adhesion or invasion stages of bacterial pathogenesis.

## Methods

### Materials

The following materials were purchased from the manufacturers indicated: heparin, heparan sulfate, chondroitin sulfate A, B and C, heparinases I and III, chondroitinase ABC, fluorescein isothiocyanate (FITC), GenElute PCR clean-up kit, phospholipase C phosphatidylinositol-specific (PI-PLC) from *Bacillus cereus*, all from Sigma-Aldrich (St. Louis, MO, USA); 2-O, 6-O and N-desulfated heparins from Amsbio (Abingdon, UK); Dulbecco’s Modified Eagle’s minimal essential medium (DMEM) and Minimum Essential Medium (MEM), fetal bovine serum, penicillin-streptomycin, and PBS-phosphate-buffered saline from Gibco-Thermo Fischer Scientific (Waltham, MA, USA); Brain-Heart Infusion broth from Pronadisa (Madrid, Spain); RNeasy Kit and RNase-Free DNase from Qiagen (Hilden, Germany); High-Capacity cDNA Reverse Transcription Kit and PowerSYBR Green PCR Master Mix from Applied Biosystems (Foster City, CA, USA). Synthetic peptides were from Abyntek Biopharma (Derio, Spain); mouse monoclonal anti-syndecan 1 (CD138) from DakoCytomation (Carpinteria, CA, USA); and rabbit anti-syndecan 2, goat anti-syndecan 3 and rabbit anti-syndecan 4 polyclonal antibodies from Santa Cruz Biotechnology (Santa Cruz, CA, USA). All other chemicals were obtained from commercial sources and were of analytical grade.

### Bacterial strains, cell lines and culture conditions

The species used in this study were the Gram-positive bacteria *Staphylococcus aureus*, *Streptococcus pyogenes*, *Streptococcus pneumoniae* and *Enterococcus faecalis*, and the Gram-negative bacteria *Escherichia coli*, *Klebsiella pneumoniae* and *Serratia marcescens*, all of which were obtained from the Hospital Universitario Central de Asturias. All the bacteria were grown in Brain-Heart Infusion broth at 37 °C in a shaking incubator, except *S. pneumoniae* which was grown in a 5% (*v*/v) CO_2_ atmosphere without shaking.

The lung cell lines used in this study were lines A549 (epithelial, ATCC® CRL-11185 ™) and MRC5 (fibroblasts, ATCC® CRL-11171 ™). The two lines were grown in DMEM and MEM, respectively, with the culture broth being supplemented with 10% (*w*/*v*) fetal bovine serum and penicillin G/streptomycin (5000 IU/ml, 5000 μg/ml). Cultures were incubated at 37 °C in a 5% (*v*/v) atmosphere.

### Fluorescein labeling

FITC labeling of bacteria was carried out using overnight cultures, which were washed four times with PBS and resuspended in a 0.1 mg/ml FITC solution to an A_600_ of 0.5; incubation in the dark at 37 °C under agitation proceeded for 1 h, and then the bacterial suspensions were centrifuged, washed 4 times with PBS to eliminate the FITC excess, and resuspended in PBS to an A_600_ of 0.5.

### Adherence assays

Adhesion of the different pathogenic bacteria to A549 and MRC5 monolayers was performed in 24-well plates grown to 70–90% confluence. The media were aspirated, and the cells washed twice with PBS and then blocked with 10% fetal bovine serum in PBS for 2 h at 37 °C in a 5% CO_2_ atmosphere. After further washing with PBS, 100 μl of FITC-labeled bacteria in 400 μl of DMEM was added and the mixture incubated for 1 h at 37 °C and 5% CO_2_. Thereafter, the wells were rinsed (4 x) with 500 μl PBS to remove unbound bacteria. At the end of the experiment, lung cells were disaggregated with 1% SDS, and the fluorescence of the pathogens attached to them was quantified in a Perkin Elmer LS55 fluorometer set at 488 nm (excitation) and 560 nm (emission). Data for the different experiments were normalized using the adhesion values without any additive or treatment, which was given the arbitrary value of 100%. Assays were performed at least in triplicate and the data are expressed as the mean ± SD.

### Inhibition of glycosaminoglycan synthesis

Cell cultures in 24 well plates at approximately 70% confluence were incubated in medium containing rhodamine B 50 μg/ml or genistein 30 μM (final concentrations) overnight at 37 °C. The cultures were washed twice with PBS, and subjected to adherence assays as described in the previous paragraph.

### Enzymatic digestion of lung cell-surface GAGs

Digestion of HS from cell cultures was achieved by incubation at 37 °C for 3 h in a 5% CO_2_ atmosphere in minimal culture medium with a mix of 500 mU/ml (final concentration) each of heparinase I and III. Digestion of CS chains was carried out by 3 h of incubation under the same conditions, but using 250 mU/ml (final concentration) of chondroitinase ABC. The digestion of both GAGs was performed through simultaneous incubation with heparinases I and III and chondroitinase ABC in the same conditions. The reactions were stopped with 2 washes in PBS buffer and the cell cultures were immediately submerged in the appropriate supplemented culture medium, and subjected to adherence assays with pathogens as described in the previous paragraph.

### Adherence inhibition assays

The effect of GAGs on adherence interference experiments was performed through the addition of either HS, CS-A, CS-B, CS-C, or a mixture of all four, at concentrations ranging between 0.01 and 400 nM to the labeled bacteria before their addition to the monolayers.

The effect of peptides which included the consensus HP binding sequences, QKKFKN and FKKKYGKS on adherence interference experiments was analyzed at a final concentration of 20 nM of each peptide. The peptides were added to the monolayers before the addition of the labeled bacteria.

The effect of the location of sulfations in HS chains on bacterial adherence was studied using native HP as control, and 2-O, 6-O and N-desulfated HPs at a final concentration of 20 nM as competitors in the adherence. The HPs were added to the labeled bacteria before their addition to the monolayers.

### RNA isolation, cDNA synthesis and qRT-PCR reactions

RNA was isolated using the RNeasy kit following the manufacturer’s specifications. Samples were subjected to treatment with RNase-free DNase during the purification process itself. The concentration of RNA obtained was determined spectrophotometrically by measuring absorbance using a Picodrop Microliter UV/Vis pectrophotometer (Picodrop Limited, UK).

cDNA synthesis was carried out using the High Capacity cDNA Transcription Kit following the manufacturer’s specifications. The reaction products were cleaned using the PCR Clean-Up GenElute kit as per the manufacturer’s instructions.

qRT-PCR reactions were carried out as previously described [17], and actin was used as a control gene to compare run variation and to normalize individual gene expression. The expression values of genes were calculated as 2^–ΔCt^ (relative to actin as the housekeeping gene).

### Antibody inhibition assays

Antibody inhibition assays were carried out in 24-well plates grown to 80% confluence. The media was aspirated, and the cells washed twice with PBS and then blocked with 10% fetal bovine serum in PBS for 2 h at 37 °C in a 5% CO_2_ atmosphere. After further washing with PBS, a mixture of anti SDC-1, anti SCD-2, anti SDC-3, and SDC-4 were diluted 1:100 in PBS, except anti-SDC2, which was diluted 1:250, and incubated for 1 h. After the treatment, adherence assays were performed as indicated above.

### Enzymatic removal of glypicans using phospholipase-C

Glypicans from cell surfaces were removed enzymatically using PI-PLC. Cells were grown in 24-well plates to 80% confluence. After washing with PBS, the cells were incubated in the absence (control) or presence of 80 mU/ml PI-PLC for 40 min at 37 °C in a 5% CO_2_ atmosphere. After the treatment, adherence assays were performed, as previously described.

### Statistical analysis

All experiments were carried out at least three times, with at least three replicates being used on each occassion. All analyses were performed using the Statistics for Windows program (Statsoft Inc.; Tulsa, OK). Mean values were compared between two samples by the Mann-Whitney *U* test and between multiple samples using the Kruskal-Walis test. The *p* value accepted as significant was *p* < 0.05. All data are presented as means ± standard errors of the means.

## Results

### GAGs are involved in the adherence of pathogenic bacteria to lung cells

**Fig. 1:**
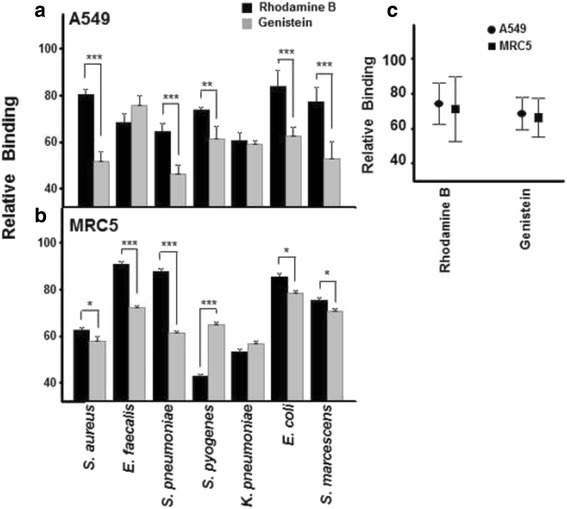
Effect of the inhibition of GAG biosynthesis on pathogen adhesion to lung cells. **a-b** Effect of treatment with either rhodamine B or genistein on binding to A549 cells **(a)** and MRC5 cells **(b)**. **c** Relative differences in mean bacterial adherence inhibition between lung cells treated with either rhodamine B or genistein. Data were normalized using values for adhesion of bacteria to non-treated cells, and spreads represent standard deviation

To analyze the possible involvement of GAGs in the adhesion of some common lung pathogens, biosynthesis inhibition experiments were performed in both lung fibroblasts and epithelial cells using two different compounds that disrupt synthesis at different levels, rhodamine B or genistein [32–35]. The cells treated with the inhibitors were exposed to previously labeled pathogens in individual experiments, resulting in a decrease in bacterial adherence in all cases, suggesting that GAGs are involved in the binding of the pathogens in both types of cell (Fig. 1a, b).

In epithelial cells, genistein displayed a statistically significant higher inhibitory effect with all the bacteria tested, with the exception of *E. faecalis* and *K. pneumoniae* (Fig. 1a). Similar results were obtained when MRC5 cells were analyzed, with genistein being the most effective at reducing the adherence of almost all bacteria, although in this case rhodamine B showed higher inhibition of *S. pyogenes* binding (Fig. 1b). When the data were analyzed as a set, however, no significant differences were observed in terms of type of inhibitor or cell line used (Fig. 1c).

### Distinct GAG species are differentially involved in the binding of pathogens to lung cells

The role played by the different molecular species of GAGs in the adherence of pathogenic bacteria was studied using two strategies: the enzymatic degradation of these molecules from the cell surface, and by binding competition experiments with commercial GAGs.

**Fig. 2:**
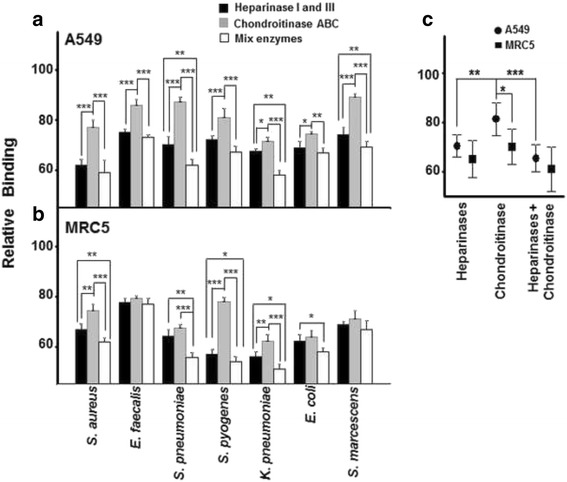
Effect of enzymatic digestion of cell GAGs in bacterial adherence to lung cells. **a-b** Effect of pre-treatment with GAG lyases: heparinase I and III, chondroitinase ABC and all enzymes simultaneously on bacterial adherence to A549 cells (**a)** and MRC5 cells (**b)**. **c** Relative differences in mean bacterial adherence between lung cells treated with each lyase individually, and with a combination of them all. Data were normalized using the values for adhesion of bacteria to non-treated cells, and spreads represent standard deviation. Statistically significant differences are denoted by * for *p* < 0.05, ** for *p* < 0.01, and *** for *p* < 0.001

The reduction of the levels of the different cell surface GAGs of the A549 and MRC5 cell lines was carried out using commercial bacterial lyases. All treatments, individual or combined, produced a decrease in bacterial adherence to lung cells (Fig. 2a, b).

In epithelial cells, the removal of both HS and CS by lyases reduced bacterial binding in all cases. The use of heparinases and the combination of all lyases showed significantly stronger effects on the adherence of all bacteria than chondroitinase alone. The combination of lyases decreased bacterial adherence to a similar extent as treatment with heparinases, except in the binding of *S. marcescens*, *S. pneumoniae* and *K. pneumoniae*, where the inhibition effect was greater (Fig. 2a). However, analyzing the averaged data, the degradation of CS produced a reduction in binding for all pathogens tested of 19% (±6.68), while treatment with heparinases resulted in an average reduction of 30% (±4.52), the difference between the two treatments being statistically significant (*p* < 0.01) (Fig. 2c). The combined digestion using both lyases decreased bacterial adherence by about 35% (±5.60) on average, which was only significantly different statistically with respect to the chondroitinase treatment (*p* < 0.001) (Fig. 2c). These data suggest that, in lung epithelial cells, both species are involved in adherence, but that HS seems to play a more crucial role.

In a similar vein, in the fibroblast cell line, digestion with bacterial lyases also caused a decrease in pathogen binding. The mixture of enzymes showed the strongest effect on binding in all cases, except *S. marcescens and E. faecalis*. Comparing the data obtained after individual use of heparinases or chondroitinase, significant differences were found in the case of the binding of *S. aureus*, *K. pneumoniae* and *S. pyogenes*, where the removal of HS showed an increased effect. For the remaining microorganisms, both types of enzymes decreased bacterial adherence to similar degrees (Fig. 2b). When average data were analyzed, treatment with chondroitinase ABC reduced adherence by 30% (±7.20), while HS treatment caused a decrease of around 35% (±7.51) (Fig. 2c). The combination of both lyases decreased adherence by more than 40%, although the differences between the effects obtained with lyases used individually and when combined are not statistically significant (Fig. 2c). It would thus seem that both types of GAG mediate in bacterial binding to a similar extent, but that the role of CS in the interaction between pathogen and fibroblast appears to be more important than in epithelial cells.

Comparing the effects of degradation of HS and CS between both lung cell lines, statistically significant differences were only observed in the treatment involving chondroitinase ABC (*p* < 0.05), which reduced adherence of bacteria more in the MRC5 than the A549 cell line.

**Fig. 3:**
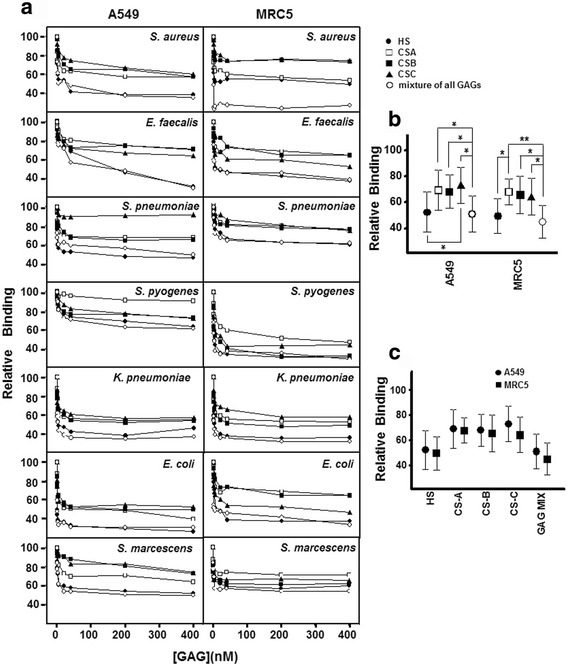
Inhibition of pathogen attachment to lung cells in the presence of different GAGs. **a** Effect on adhesion to A549 cells and to MRC5 cells of bacteria: *Staphyloccocus aureus*, *Enteroccocus faecalis*, S*treptococcus pneumoniae*, *Streptoccocus pyogenes*, *Klebsiella pneumoniae*, *Escherichia coli*, and *Serratia marcescens* in the presence of different concentrations of each GAG individually and with a combination of all. **b** Comparative effect of the various individual GAG species and a mixture containing all of them on mean bacterial adherence to the different lung cell lines. **c** Comparison between lung cells with respect to the effect of the various different GAG species and a mixture of them all on mean bacterial adherence. Data are shown normalized to a control represented by binding in the absence of interfering molecules. The spreads represent standard deviation. Statistically significant differences are denoted by * for *p* < 0.05, and ** for *p* < 0.01

To further establish the influence of the several species of GAGs as bacterial receptors in lung cells, diverse adherence interference experiments were performed using commercial HS, CS-A, CS-B, CS-C and an equimolar mixture of each of them, as described in the Methods section. The presence of GAG molecules decreased the binding of all bacteria tested in both the A549 line (Fig. 3a) and the MRC5 lines (Fig. 3b). Furthermore, in both lines adherence was reduced in a dose dependent manner.

In epithelial cells, when commercial GAGs were used individually, HS was the most effective interfering molecule. However, the role played by each type of CS varied, depending on the pathogen tested (Fig. 3a). When a mixture of all the GAGs was used, the effect obtained was similar to when using HS, with the exception of *S. pneumoniae*, which at low concentrations showed greater inhibition of adherence by HS (Fig. 3a). While no statistically significant differences between the mixture of GAGs and HS were found, significant differences were found between HS and CS-C (*p* < 0.05), as well as between the mixture of all GAGs and each type of CS individually (*p* < 0.05) (Fig. 3c).

In MRC5 cells, bacterial adherence was also reduced by the different species of GAGs used individually and by the mixture of GAGs (Fig. 3a). Comparing the individual effects of each GAG type, HS displayed the highest inhibition effect on adherence in all pathogens except *S. marcescens,* where CS-B showed similar values. As in the epithelial cells, the effect of the different CS species was dependent on the bacteria involved. Moreover, the effect of the equimolar mixture of GAGs was similar to HS, the only exception being *S. aureus,* where the combination of GAGs inhibited adherence more effectively (Fig. 3a). Taken together the results evidence that both, HS and the mixture of GAGs, showed the highest inhibitory effect, and no statistically significant difference was found between them (Fig. 3b). However, the mixture of GAGs displayed significant differences with every individual CS: CS-A (*p* < 0.01), CS-B (*p* < 0.05) and CS-C (*p* < 0.05); significant differences were also found between HS and CS-A (*p* < 0.05) (Fig. 3b).

In contrast, when the effects observed for each interfering molecule individually were compared to the impact of the combination of all of them were compared, no differences between the two types of lung cell were detected (Fig. 3c).

### Differential interference of peptides with HP binding sequences in the binding of pathogenic bacteria to lung cells

**Fig. 4:**
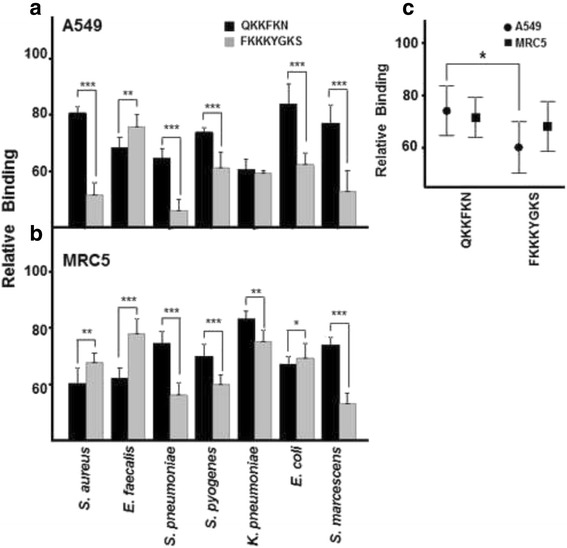
Effect of peptides with heparin binding sequences on pathogen attachment to lung cells. The effects of QKKFKN and FKKKYGKS (20 nM final concentration) on bacterial binding to A549 cells (**a**) and to MRC5 cells (**b**). **c** Relative differences in mean bacterial adherence inhibition between lung cells in competition with each individual peptide. Data are shown normalized to the control represented by binding in the absence of interfering molecules. The spreads represent standard deviation. Statistically significant differences are denoted by * for *p* < 0.05

Two peptides which include consensus HP binding sequences of different lengths, QKKFKN and FKKKYGKS, were designed to examine their influence on bacterial binding to lung cells. The effect of both peptides was first calibrated in our laboratory in adherence interference experiments using *S. aureus* and *S. marcescens*. The influence of both QKKFKN and FKKKYGKS was dose-dependent, resulting in a reduction in binding up to concentrations of 10–20 nM, diminishing their effects at higher concentrations (data not shown). Consequently, a concentration of 20 nM of each peptide was selected for subsequent adhesion assays. The use of either of the peptides caused a decrease in the binding of every pathogen tested in both types of lung cell (Fig. 4a, b).

In the A549 line, despite both peptides reducing bacterial binding, the long peptide was significantly more efficient in all cases except *K. pneumoniae*, where values were similar to those for the short peptide, and for *E. faecalis* binding where the short peptide showed the greater effect (Fig. 4a). Average values for the reduction of adherence for the long peptide were 40% (±9.80), while for short peptide they were 26% (±9.46). This observed difference between the effect of the two molecules, was analyzed and found to be significant only in epithelial cells (*p* < 0.05) (Fig. 4c).

In contrast, in fibroblasts, the effect of individual peptides depended on the pathogen in question. The long peptide had a significantly stronger effect on reducing adherence of *S. pneumoniae*, *S. marcescens, K. pneumoniae* and *S. pyogenes*, whereas the short peptide was more efficient in decreasing the binding of *E. faecalis*. Both peptides decreased adherence to a similar extent for *E. coli* and *S. aureus* (Fig. 4b). Considering all the results together, adherence was reduced by 28.5% (±7.67) with the short peptide and 68.06 (±9.53) with the long peptide, though the difference was not statistically significant (Fig. 4c).

Neither were significant differences found when comparing the results of each peptide between the two cell types (Fig. 4c).

### Differential involvement of cell-surface HSPGs in the binding of pathogenic microorganisms to lung cells

To determine which species of HSPGs were expressed in both epithelial and fibroblast cell membranes, a transcriptome study of the genes encoding the core proteins of all the syndecan and glypican members was performed.

**Fig. 5:**
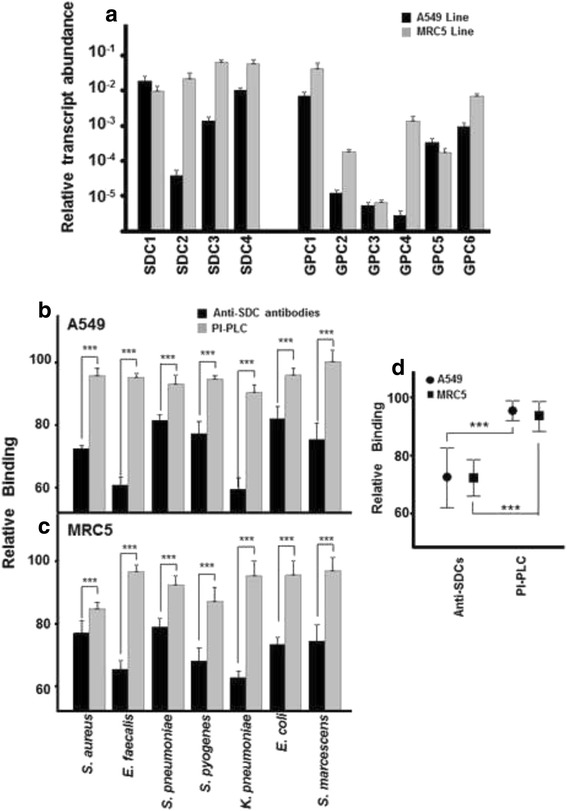
Involvement of cell surface HSPGs in the binding of pathogens to lung cells. **a** Differential transcription of syndecans and glypicans in lung cells; values on the Y-axis are represented on a logarithmic scale. **b-c** Effect of treatment with anti-SDC antibodies and with PI-PLC on pathogen attachment to A549 cells (**b)** and MRC5 cells (**c)**. **d** Relative differences in mean bacterial adherence inhibition between lung cells treated with anti-SDC antibodies and with PI-PLC. Data are shown normalized to the control represented by binding in the absence of treatment. The spreads represent standard deviation. Statistically significant differences are denoted by *** for *p* < 0.001

In A549 cells, transcripts for the four SDC isoforms were detected with different levels of expression, isoform 1 and 4 being the most strongly expressed, and isoform 2 showing the lowest level of expression. Regarding transcripts for GPCs, all six isoforms were also detected, but the magnitude of expression levels found varied widely. *GPC-1* was the most abundant, with *GPC-5* and *-6* transcripts being expressed at levels about one order of magnitude, and those of *GPC-2*, *−3* and *−4* around three orders of magnitude lower than levels *GPC-1* (Fig. 5a). In contrast, the arrangement of cell surface HSPG expressions in MRC5 cells was quite different: the four syndecan isoforms all appeared highly transcribed, and while the levels of the different glypican isoforms varied between themselves by about three orders of magnitude, similar to in the A549 cells, the pattern of expression was quite distinct, particularly for *GPC-2* and *-4*, which expressed at higher levels (Fig. 5a).

To analyze the possible involvement of these families of HSPGs in pathogen binding to lung cells, different adherence essays were performed. To establish the role of GPCs in the binding of pathogens, they were removed from the cell surface using PI-PLC, an enzyme that cleaves their GPI-anchor. The results showed that this treatment only slightly decreased bacterial adherence in both A549 cells (Fig. 5b), and MRC5 cells (Fig. 5c), for all pathogens. In epithelial cells, binding was reduced by 4% (±3.52), and, in fibroblasts by 6% (±5.19). Comparing the average values obtained in each cell line, no great differences were detected between epithelial cells and fibroblasts. (Fig. 5d).

The involvement of SDCs in bacterial adherence was analyzed by blocking experiments using a combination of specific antibodies against the four isoforms of these molecules. In all cases, in both epithelial and fibroblast cells, this treatment decreased bacterial binding (Fig. 5b, c), by 27% (±10.32) in A549 cells and 28% (±6.31) in MRC5 cells (Fig. 5d), the difference not being significant.

The analysis of the differences between PI-PLC and anti-SDC treatments on each bacterium followed a general pattern, which revealed the significantly higher involvement of SDCs in binding to epithelial cells (Fig. 5b). In terms of adherence to fibroblasts, the differences between the two treatments were statistically significant for all bacteria (Fig. 5c).

Additionally, when all the data were analyzed, the differences between PI-PLC and anti-SDC treatments on bacterial adherence were significant for both cell lines (*p* < 0.001) (Fig. 5d).

### Influence of specific N- and O- sulfations on bacterial adherence to lung cells

**Fig. 6:**
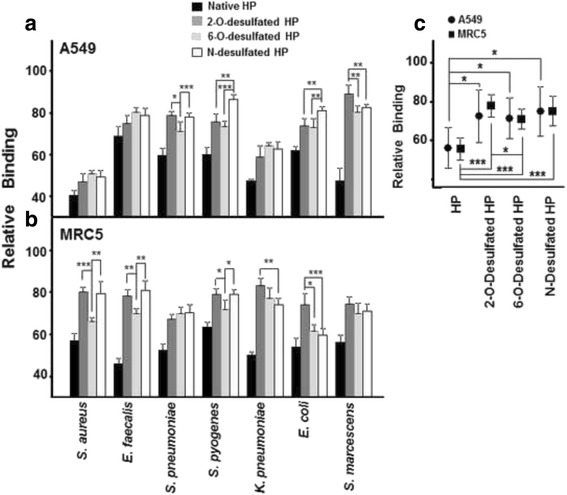
Influence of specific N- and O- sulfations on pathogen adherence to lung cells. **a-b** Inhibition of the binding of microorganisms to A549 cells (**a**) and MRC5 cells (**b**) by the presence of native heparin, 2-O desulfated heparin, 6-O-desulfated heparin and N-desulfated heparin. **c** Comparative effect of the different heparins on mean bacterial adherence to lung cells. Data are shown normalized to the control represented by binding in the absence of interfering molecules. The spreads represent standard deviation. The differences between native HP and desulfated HPs are not shown in the figure, being all of them significant. Statistically significant differences are denoted by * for *p* < 0.05, and *** for *p* < 0.001

To analyze the influence of sulfation at specific positions on the disaccharide unit of HS chains on interaction with bacteria, a variety of adherence interference experiments were performed using 2-O, 6-O and N- desulfated HPs and compared to normal HP as control. For all the pathogens tested, the presence of any heparin-derived molecule diminished binding, both to A549, and to MRC5 cells, although the results varied depending on the pathogen (Fig. 6).

In epithelial cells, desulfated HPs were less effective in inhibiting the adhesion of all bacteria tested than fully sulfated HP, although the scale of the effect varied depending on the specific microorganism analyzed (Fig. 6a). Analyzing the effects of desulfated HPs for each bacterium the results showed similar values in *S. aureus*, *K. pneumoniae* and *E. faecalis*. In the adherence of *E. coli* and *S. pyogenes* 2 and 6-O-desulfated HP showed significantly higher effects than N-desulfated HP whereas, 6-O- and N-desulfated HP affected the bonding of *S. marcescens* even more strongly, and 6-O-desulfated HP decreased the adherence of *S. pneumoniae* more than other desulfated HPs did (Fig. 6a). Pooling all the data, no significant differences were observed between in inhibition values for the three forms of desulfated HP tested, although each showed significant differences when compared to normal HP (*p* < 0.05) (Fig. 6c).

In fibroblasts, binding was also inhibited by the presence of each of the tested molecules, but in this case the effect on the different pathogens was more homogeneous than in epithelial cells. As in the A549 cell line, both when analyzing the data of each bacterium individually, and when the set of all bacteria were considered together, native HP displayed the highest inhibitory effect on adhesion in all cases, showing statistically significant differences compared with each of the other desulfated-HPs (*p* < 0.001) (Fig. 6b, c). Comparing the individual effect of each desulfated HP, very different results were obtained depending on bacterium involved. While no significant differences were found between the effects of these molecules on the adherence of *S. pneumoniae* and *S. marcescens*, 6-O-desulfated HP showed the highest effect on binding for *S. pyogenes*, *S. aureus* and *E. faecalis*. 6-O- and N-desulfated HP reduced bonding of *E. coli* more significantly, and N-desulfated HP showed significant differences with 2-O-desulfated HP in the case of *K. pneumoniae* (Fig. 6b). However, regarding the average data, in these cells differences dependent on the type of sulfation were detected, with 6-O-desulfated HP able to compete more effectively than 2-O-desulfated HP (*p* < 0.05), while N-desulfated molecules produced more heterogeneous results (Fig. 6c).

## Discussion

PGs are involved in many infectious processes, especially by their GAG moieties, which act as receptors for the adherence and attachment of many pathogenic microorganisms, including some which affect the respiratory tract [24, 26]. Lung epithelial cells are in contact with the environment, creating a barrier against pathogens, along with the other passive and active defense mechanisms. Lung fibroblasts are found in the lower layers, and are thus less exposed to environmental factors and microorganisms. It would therefore be expected that both cell types present a distinct profile of PGs, including GAGs, with different structures, which would affect their interaction with pulmonary pathogens. The characterization of GAG-pathogen interactions could allow new and more efficient infection avoidance strategies to be developed via preventing adhesion.

To determine the involvement of PGs, and especially their GAG moieties, in bacterial attachment to A549 and MRC5 cell lines, their levels were reduced by the use of two specific inhibitors of their biosynthesis: rhodamine B or genistein, and the effect on pathogen adherence analyzed. These treatments resulted in a significant decrease in bacterial adherence in all cases, indicating that the lung pathogens use, at least in part, GAGs as receptors for adhesion. We have recently published a study of bacterial adherence to corneal epithelial cells, where it was noted that treatment with rhodamine B or genistein had a different effect on bacterial adherence depending on the Gram nature of the microorganism in question. Specifically we found that in Gram-positive bacteria binding was most affected by rhodamine, while genistein more strongly reduced Gram-negative adhesion [36]. Interestingly, the current study was unable to determine the existence of such patterns in the adherence of microorganisms to lung cell lines, being the behavior observed dependent, for each specific cell line, of the bacterial species analyzed.

The results observed may be due to the fact that these molecules affect the biosynthesis of GAGs at different levels. Rhodamine B is thought to inhibit chain elongation, acting as a nonspecific inhibitor that reduces GAG synthesis in a range of cells, and produces reduced lysosomal GAG storage in some types of mucopolysaccharidosis [32, 33]. The isoflavone genistein inhibits the kinase activity of epidermal growth factor receptor, which is required for the full expression of genes coding for enzymes involved in GAG production [34], although it has been described that the effect of this molecule on the biosynthesis of GAGs is strongly dependent on their type and localization [35]. Each bacterium has different types of adhesins to bind to the chains of GAGs of lung cells, and each one of these molecules has a variable binding specificity, which can be determined by various factors, such as the length of the chains, which would be affected by rhodamine B, or by the structure of the GAG chains, which can be altered by treatment with genistein.

The involvement of GAG species in bacterial adherence to lung cells was analyzed by reducing the levels of GAG chains by means of enzymatic degradation using bacterial lyases. The results show that both types of GAG are involved in the binding of bacteria to lung cells. In A549 cells, heparinase treatment significantly decreased adherence relative to chondroitinase treatment, but not compared with simultaneous digestion of HS and CS chains, suggesting that HS is the main species involved in bacterial adherence, and that there is no additive effect in the presence of both molecules. In MRC5 cells, HS also seems to be the principal molecule involved in adherence, but in this case, chondroitinase treatment produced a similar decreases as HS with respect to certain bacteria, suggesting that in this cell line CS has a greater involvement in bacterial adhesion. The differences observed in chondroitinase treatment for both cell lines were statistically significant, which could be a reflection of the existence of differences between A549 and MRC5 cell lines as regards PG composition and structure of GAG chains, considering that the expression pattern of GAGs varies depending on cell type and their physiological state.

The participation of the predominant GAG species present on the cell surface in the adhesion of pathogens was also studied by analyzing their ability to compete in adherence interference experiments. HS showed the highest inhibitory ability, suggesting that it constitutes the predominant cell surface receptor for all the bacteria analyzed, although in the case of binding of *S. marcescens* to fibroblasts, CS-B showed an effect comparable to HS. There was no clear pattern in the inhibition produced by the diverse types of CS tested in both epithelial cells and fibroblasts depending on the bacteria tested. When the inhibitory effect of an equimolar mixture of all the GAG species was analyzed, in most cases results were similar to HS used alone, suggesting, again, that this species is the main cell surface receptor. An interesting exception to the previous conclusion was the binding of *S. aureus* to fibroblasts, where the effect of the mixture was considerably higher than that produced by HS alone, suggesting that in this cell type different molecular species cooperate in the binding of the pathogen.

All the results obtained in this work suggest HS to be the main mediator in bacterial cell adhesion, which fits with the fact that it is the most widespread GAG on cell surfaces [12, 13]. A large number of bacteria use this molecule as a receptor for adhesion to different tissues, including *Helicobacter pylori*, *E. faecalis*, *Neisseria meningitidis*, *Pseudomonas aeruginosa*, and *S. aureus*, among others [24]. Other GAGs are also able to act as receptors for microorganisms, such as *Borrelia burgdorferi*, which uses a variety of these molecules as receptors depending on the host cells; HS to bind to endothelial cells and CS-B and HS to glial cells [37]. HS and CS-B chains also mediate in *Chlamydia trachomatis, S. pyogenes* binding [24]. Both HS and CS-B share a feature in their chemical structure, variable proportions of the C-5 epimer of glucuronic acid, the iduronic acid, providing the molecule with more flexibility [38]. Some of the most common pathogenic bacteria in the respiratory tract also use GAGs, mainly HS, for attachment, such as *S. pneumoniae* [39], 75% of nontypeable *Haemophilus influenzae* [40], *Chlamydia pneumoniae*, and *P. aeruginosa* on basolateral epithelial surfaces [24].

HS has a complex domain structure, consisting of highly sulfated NS-domains interspaced with poorly sulfated NA-domains. Most interactions between proteins and HS occurs mainly through NS-domains, either by electrostatic interactions or by the specific recognition of sulfated sequences in saccharide chains. Most heparin-binding proteins have clusters of basic amino acids alternating with hydropathic residues that have been shown to be required for binding [41]. In accordance with these consensus sequences, two peptides, FKKKYGKS and QKKFKN, were designed in order to figure out their role as competitors for bacterial attachment and, consequently, their ability to mimic different binding motives present on the bacterial adhesins. Both peptides partially inhibited adhesion in both cell lines, and in most cases the longer peptide was more efficient. However, the pattern observed changed depending on the bacteria and, particularly, on the pulmonary cell type, suggesting that different bacterial adhesins with different HP binding sequences are mediating in interaction with lung cells [8, 42, 43].

Generally, HS chains occur as HSPGs, and two gene families, SDCs and GPCs, account for most cell surface HSPGs. Some previous studies have related specific species of cell surface HSPGs with the adhesion of and colonization by certain pathogens [7, 14, 42]. Since HSPGs are expressed in varying amounts depending on the tissue, we quantified their transcript levels in both lines of pulmonary cell. In the MRC5 line, transcripts for the four SDCs could be detected at similar levels, but in the A549 line isoforms 1 and 4 were much more abundant. With respect to GPCs, the six isoforms showed large differences in their transcript levels, dependent on the cell type, although isoform 1 was the most abundant in both cases. The enzymatic removal of GPCs from the cell surface did not show any great effect, while blocking experiments with a combination of anti-SDC specific antibodies resulted in a decrease in adherence in both cell lines, suggesting that SDCs are involved in bacterial attachment to lung cells.

Similar results were found in a study of bacterial adhesion to corneal epithelial cells, where all the isoforms of SDCs participate cooperatively in the attachment of bacteria, indicating that they play a far more important role in this process than GPCs [36]. The involvement of SDCs in bacterial adherence has been described for different pathogens such as *Streptococcus agalactiae, Listeria monocytogenes* and *S. aureus,* which interacts with syndecan-1 for adherence to cells; and *Neisseria gonorrhoeae,* whose adhesin OpaA uses SDC-1 and -4 as receptors [44–46]. There is little information on the involvement of GPCs in infectious processes, an interesting exception being the case of the intracellular pathogen *Chlamydophila pneumonia*, which is able to use a variety of cell-type specific binding mechanisms, e.g. it uses GAG chains to bind to epithelial cells, but in Jurkat lymphoid cells, which only express GPC-1, it uses a GAG-independent mechanism to bind. However, it is known that GPCs are involved in other non-infectious diseases, including tumoral processes, neurological syndromes and disorders, and prion diseases [22, 47–50].

In some pathogens, it has been described that specific patterns of sulfation are required for adherence; this is the case for *C. trachomatis*, whose binding to HeLa 229 cells is inhibited by 2-O desulfated HP [51], certain viruses such as the hepatitis E virus, which interacts with 6-O-sulfated HS [52], and baculovirus, whose entrance is promoted by 6-O-and N-sulfated HS [53]. To investigate the importance of specific sulfated positions of the HS disaccharide unit on the binding of bacteria to lung cells, the effect of native HP compared to 2-O, 6-O and N-desulfated HPs in competition adherence assays was analyzed.

All desulfated HPs showed less inhibitory capacity than the native HP, which points to the importance of sulfation in adherence. However, the effect was slightly different between the two cell lines; while in the epithelial cells results varied widely depending on the pathogen analyzed, and no significant differences between the different desulfated heparins were observed, in fibroblasts results were more homogeneous, and sulfation in C2 of uronic acid showed a greater effect on competition sulfation in C6 of the glucosamine residue. These data suggest that binding involves different sulfated sequences in each cell type, which could also relate to the use of different bacterial adhesins in each case.

## Conclusions

In summary, this work describes that GAGs seem to be implicated in the binding of different pathogenic bacteria both to epithelial cells and to lung fibroblasts. HS seems to be the principal GAG species involved, especially when conjugated in SDCs. But the molecular features of the binding seems to be dependent on the bacteria and, particularly, on the type of pulmonary cell involved, which appear to involve different HS sulfation patterns and different bacterial adhesins. Increased understanding of the relation between bacteria and their receptors, especially HS, and the influence of certain details of its structure, may lead to opportunities for developing innovative and selective therapies in the future. From this perspective, the structure of the GAG molecules and their implication in interactions with microorganisms deserve further investigation into their possible therapeutic role in a variety of pulmonary infections.

## Abbreviations

CS: Chondroitin sulfate
ECM: Extracelullar matrix.
FITC: Fluorescein isothiocyanate.
GAG: Glycosaminoglycan.
GPC: Glypican.
HP: Heparin.
HS: heparan sulfate.
HSPG: Heparan sulfate proteoglycan.
PG: Proteoglycan.
PI-PLC: Phospholipase C phosphatidylinositol-specific.
SDC: Syndecan.
